# Increases in intracellular calcium via activation of potentially multiple phospholipase C isozymes in mouse olfactory neurons

**DOI:** 10.3389/fncel.2014.00336

**Published:** 2014-10-21

**Authors:** Steven A. Szebenyi, Tatsuya Ogura, Aaron Sathyanesan, Abdullah K. AlMatrouk, Justin Chang, Weihong Lin

**Affiliations:** Department of Biological Sciences, University of Maryland Baltimore CountyBaltimore, MD, USA

**Keywords:** phospholipase C isozyme, calcium imaging, olfactory sensory neuron, real-time qPCR, RNA *in situ* hybridization

## Abstract

Phospholipase C (PLC) and internal Ca^2+^ stores are involved in a variety of cellular functions. However, our understanding of PLC in mammalian olfactory sensory neurons (OSNs) is generally limited to its controversial role in odor transduction. Here we employed single-cell Ca^2+^ imaging and molecular approaches to investigate PLC-mediated Ca^2+^ responses and its isozyme gene transcript expression. We found that the pan-PLC activator m-3M3FBS (25 μM) induces intracellular Ca^2+^ increases in vast majority of isolated mouse OSNs tested. Both the response amplitude and percent responding cells depend on m-3M3FBS concentrations. In contrast, the inactive analog o-3M3FBS fails to induce Ca^2+^ responses. The m-3M3FBS-induced Ca^2+^ increase is blocked by the PLC inhibitor U73122, while its inactive analog U73433 has no effect. Removal of extracellular Ca^2+^ does not change significantly the m-3M3FBS-induced Ca^2+^ response amplitude. Additionally, in the absence of external Ca^2+^, we found that a subset of OSNs respond to an odorant mixture with small Ca^2+^ increases, which are significantly suppressed by U73122. Furthermore, using reverse transcription polymerase chain reaction and real-time quantitative polymerase chain reaction, we found that multiple PLC isozyme gene transcripts are expressed in olfactory turbinate tissue in various levels. Using RNA *in situ* hybridization analysis, we further show expression of β4, γ1, γ2 gene transcripts in OSNs. Taken together, our results establish that PLC isozymes are potent enzymes for mobilizing intracellular Ca^2+^ in mouse OSNs and provide molecular insight for PLC isozymes-mediated complex cell signaling and regulation in the peripheral olfactory epithelium.

## INTRODUCTION

Phospholipase C (PLC) is one of the most common and important enzymes in cell signaling. Activation of PLC and its associated pathways by cell surface receptors including G-protein coupled receptors and receptor tyrosine kinases, enable cells to react to numerous internal and external signaling molecules, modulators, and stimuli (Suh et al., 2008; Yang et al., 2013). Upon activation, PLC catalyzes the hydrolysis of phosphatidylinositol 4, 5-bisphosphate (PIP_2_) to produce two second messengers: diacylglycerol (DAG) and inositol 1, 4, 5-trisphosphate (IP_3_), both of which are capable of triggering a variety of cellular events (Majerus et al., 1986; Singer et al., 1997; Rhee, 2001). In the olfactory system, the roles of PLC and its associated second messengers depend on species and sub-olfactory organs. While it is well known that the IP_3_-mediated pathway plays an important role in olfactory transduction of invertebrates and aquatic vertebrate animals (Fadool and Ache, 1992; Miyamoto et al., 1992; Schild et al., 1995; Bruch, 1996; Munger et al., 2000; Sansone et al., 2014), in rodent olfactory transduction, the role of the PLC pathways remains unclear because of inconsistent results in the literature. It has been reported that certain odors stimulate IP_3_ production (Boekhoff et al., 1990; Breer et al., 1990; Liu et al., 2006; Klasen et al., 2010) and that IP_3_ reportedly activates membrane ion channels in olfactory sensory neurons (OSNs; Restrepo et al., 1992; Okada et al., 1994; Lischka et al., 1999; Kaur et al., 2001; Liu et al., 2006). However, genetic knockout or pharmacological inhibition of the cAMP pathway, which is the canonical olfactory transduction pathway present in a majority of rodent OSNs, abolishes or severely suppresses odor responses to a wide range of odorants, including those stimulating IP_3_ production (Brunet et al., 1996; Belluscio et al., 1998; Chen et al., 2000; Wong et al., 2000; Zhao and Reed, 2001; Lin et al., 2004; Ma, 2012). Adding to the complexity, we previously showed that PLC inhibitor U73122 suppresses pheromone-induced electro-olfactogram (EOG) responses in the main olfactory epithelium (MOE) of wild type mice after the cAMP pathway is blocked (Lin et al., 2004). Furthermore, cyclic nucleotide gated channel A2 subunit (CNGA2) knockout mice are able to detect certain odorants behaviorally in experiments using an automated olfactometer system (Lin et al., 2004; Clevenger and Restrepo, 2006). These results indicate a role of PLC, although it is not dominant, in mammalian odor transduction.

Knowledge of PLC beyond olfactory transduction in the peripheral olfactory system is largely missing. To date, there is no report on direct activation of PLC in OSNs to evaluate its influence on intracellular Ca^2+^, despite one of the major functions of the PLC pathways is to mobilize intracellular Ca^2+^ to participate in and modulate cellular activity. Also, there is only scattered information about the expression of PLC isozymes (Liu et al., 2006). In mammals, 13 PLC isozymes have been identified and are divided into six groups: PLC-β, -γ, -δ, -ε, -ζ, and -η based on their amino acid sequences (Gresset et al., 2012). These isozymes, together with their second messenger DAG and IP_3_-mediated pathways, play essential roles in a variety of cellular functions primarily via protein kinase-mediated phosphorylation and elevation in intracellular Ca^2+^ levels (Rebecchi and Pentyala, 2000; Suh et al., 2008).

To increase our understanding of the PLC functions in the olfactory system, we first isolated OSNs from the MOE and surveyed PLC activity using a pan-PLC activator and single-cell intracellular Ca^2+^ imaging. Because the use of the activator in OSNs has not been reported, we conducted a series of stringent experiments to demonstrate its specificity in OSNs. Second, we identified the primary Ca^2+^ source for PLC-mediated Ca^2+^ increase. Third, we determined a small portion of odor-induced Ca^2+^ increases is from PLC-mediated internal Ca^2+^ release. Fourth, using reverse transcription polymerase chain reaction (RT-PCR) and real-time quantitative polymerase chain reaction (qPCR), we identified multiple PLC isozymes gene transcripts in RNA extracted from the olfactory turbinate tissue and quantified their expression levels. Finally, using RNA *in situ* hybridization (RISH), we show transcript expression of PLC isozyme β4, γ1, and γ2 in OSNs. Altogether, our data provides physiological and molecular insight for future studies investigating the roles of PLC and its second messenger-mediated signaling pathways in mammalian OSNs.

## MATERIALS AND METHODS

### ANIMALS

Adult male and female C57BL/6 background mice of 2–6 months old were used. These mice were in-house bred and were offspring of a transgenic mouse line generated originally in Dr. Robert R. Margolskee’s laboratory, in which the promoter of transient receptor potential channel M5 (TRPM5) drives the expression of GFP (Clapp et al., 2006). We used these mice because of tissue-sharing with other experiments to minimize the number of animals used. All animal care and procedures were approved by the Animal Care and Use Committees of University of Maryland, Baltimore County.

### SOLUTIONS AND CHEMICALS

Chemicals for solutions were purchased from Sigma-Aldrich (St. Louis, MO, USA). Normal bath solution (Tyrode’s saline) contained (in mM): 140 NaCl, 5 KCl, 10 *N*-2-hydroxyethylpiperazine- *N* = -2-ethanesulfonic acid buffer (HEPES), 1 MgCl_2_, 3 CaCl_2_, 10 Na pyruvate, and 10 D-glucose (pH 7.4 with NaOH). For nominal Ca^2+^ free bath solution, CaCl_2_ was omitted from the normal Tyrode’s saline. In some experiments as specified in the text, 1, 2-bis(o-aminophenoxy)ethane-*N,N,N*′,*N*′-tetracetic acid (BAPTA) was added to a final concentration of 210 μM or 5 mM (for odorants stimulation experiments). PLC activator 2,4,6-trimethyl-*N*-(*m*-3-trifluoromethylphenyl) benzenesulfonamide (m-3M3FBS) and its inactive analog o-3M3FBS (Tocris Bioscience, Minneapolis, MN, USA) were dissolved in dimethylsulfoxide (DMSO) to a stock concentration of 25 mM and freshly diluted immediately before each experiment in bath solution to a final concentration of 15 or 25 μM. The PLC inhibitor U73122 and its inactive derivative U73433 (Tocris) were dissolved in DMSO in stock and diluted in bath solution to a final concentration of 5 or 10 μM. The adenylyl cyclase activator forskolin (EMD Millipore) was dissolved in DMSO in stock and diluted in bath solution to a final concentration of 3 μM. The final concentration of <0.1% DMSO did not induce changes in Ca^2+^ levels in our Ca^2+^ imaging experiments. Odor chemicals were purchased from Sigma-Aldrich at the highest purity available. The odor mix used included pentanol, phenethyl alcohol, pentyl acetate, citral, geraniol, isobutyraldehyde, octanal, decanal, hexanal, *trans*-cinnamaldehyde (10 μM each). Odorants were made by dilution with vigorous vortexing in Tyrode’s saline from 10 mM stock solution stored in 20°C.

### ENZYMATIC ISOLATION OF OSNs

The method of OSN isolation was adapted from our previous study (Ogura et al., 2011). Briefly, mice were euthanized by CO_2_ asphyxiation followed by cervical dislocation and exsanguination through an open heart. The head skin was removed and the nose was split from the midline. The nasal turbinates were dissected and placed in Ca^2+^- Mg^2+^- free Tyrode’s saline containing ∼2.5–4 U/ml of papain (Worthington, Lakewood, NJ, USA) and 2 mM cysteine for 2.5–3.5 min at room temperature. Gentle pipetting at the end of enzyme incubation was applied to facilitate cell dissociation. The supernatant was transferred to an O-ring chamber on a cover slip pre-coated with concanavalin A (Sigma).

### Fura-2 RATIO Ca^2+^ IMAGING

Intracellular Ca^2+^ levels of OSNs were monitored using Ca^2+^ sensitive dye Fura-2 as described in our previous publication (Ogura et al., 1997, 2010, 2011). Briefly, cells were loaded with 2 μM Fura-2 AM (Molecular Probes) for 20–25 min. A pair of Fura-2 fluorescence images were captured every 3 s at 340 and 380 nm excitation lights using an inverted microscope (Olympus IX71) equipped with a UAPO/340 40x objective lens, a Hamamatsu CCD camera, a Sutter LS Xenon light source/filter changer controlled by Imaging Workbench software version 6 (INDEC BioSystems, Santa Clara, CA, USA). We measured Ca^2+^ levels as ratio of fluorescence values from 340 nm excitation/380 nm excitation light images. A change in the intracellular Ca^2+^ levels (ratio of F340/F380) is considered to be a stimulus-induced response if the peak value of the change during stimulation was greater than 5% of the resting level and within 30 s after stimulation, The resting Ca^2+^ level was obtained by averaging 10 data points (3 s each) before applying the stimulus in each cell tested or using the value of the first point before an assumed response if the baseline was stable. In figures containing Ca^2+^ traces, the vertical scale bar stands for a 50% change in the Ca^2+^ level measured from the resting Ca^2+^ level of the recorded region of the same cells.

### REVERSE TRANSCRIPTION POLYMERASE CHAIN REACTION

#### Primer design

Oligonucleotide primers were designed against each of the PLC isozymes using NCBI Primer BLAST (Ye et al., 2012) or the primer sequences were obtained from the Harvard PrimerBank (Spandidos et al., 2010). Primers were custom-made by Life Technologies (Carlsbad, CA, USA). Individual isozyme NCBI GI numbers, primer sequences, expected amplicon sizes, targeted splice variants and PrimerBank ID are listed in **Table 1**.

**Table 1:** Oligonucleotide primer sequences for individual PLC isozymes and their applications (RT-PCR and/or qPCR).

Gene symbol	NCBI GI no.	Primer sequence (5′ = Forward; 3′ = Reverse)	Expected amplicon size (bp)	Applications
*Plcb1* (β1)	224967067	5′: GCCCCTGGAGATTCTGGAGT 3′: GGGAGACTTGAGGTTCACCTTT	124	RT-PCR, qPCR (PrimerBank ID: 4099293a1)

*Plcb2* (β2)	61676178	5′: TGGATGTCACGAGTATCCGAG 3′: GTTTCTGGCTCTTGGGTATCTTT	798	RT-PCR

		5′: AGGATAGCTGTGATGGAAGAAGG 3′: GCCCAGGTGTCAGGTATGTAG	176	qPCR (PrimerBank ID: 27370658a1

*Plcb3* (β3)	118130639	5′: CTGCCGCTCTATCTTTGGGG 3′: GCCGATGTCGCTTCTTATTCTTC	134	RT-PCR, qPCR (PrimerBank ID: 1246801a1)

*Plcb4* (β4)	118130923	5′: CAAGGGAGGCCGAGTTGATT 3′: GGTCAGGCAGGATCACCTTT	428	RT-PCR

		5′: GGACAAGTGCTAGAATGTTCCC 3′: GAAGCCGATATTCACCAGATCC	165	qPCR (PrimerBank ID: 29293803a1)

*Plcg1* (γ1)	118129851	5′: CGCTGCATTGAGTTGGACTG 3′: TTCGTCTGTGGAACAGGCTC	899	RT-PCR

		5′: ATCCAGCAGTCCTAGAGCCTG 3′: GGATGGCGATCTGACAAGC	105	qPCR (PrimerBank ID: 1246803a1)

*Plcg2* (γ2)	26986602	5′: GTGGACACCCTTCCAGAATATG 3′: ACCTGCCGAGTCTCCATGAT	137	RT-PCR, qPCR (PrimerBank ID: 26986603a1)

*Plcd1* (δ1)	118130605	5′: CAGCTCGTGGCGTAGAGAAC 3′: CCTGAATGTCCTCGATGGAGAA	122	RT-PCR, qPCR (PrimerBank ID: 9790167a1)

*Plcd3* (δ3)	258645147	5′: GGCTACGGGCACTGAAGAAG 3′: GCTGCACGAAGAATATGTGCTT	198	RT-PCR, qPCR (PrimerBank ID: 22779905a1)

*Plcd4* (δ4)	125347170	5′: GAAGGTTATGAAGTGTCCGATGT 3′: AACTGCTTTGACAAGAGAATGGA	102	RT-PCR, qPCR (PrimerBank ID: 22507345a1)

*Plce1* (ε1)	134053942	5′: TTCGTCGAGCTGTTCAAATCA 3′: GCAGGGTACAGAGTAGATGTCA	81	RT-PCR, qPCR (PrimerBank ID: 26330738a1)

*Plch1* (η1)	295148203	5′: ACTACCAGTCTGAAGGGCGA 3′: GCTTCCTATTCAGATCCAGACAG	966	RT-PCR

*Plch2* (η2)	294862234	5′: GTGGACGACAACGGATTCAAC 3′: CTTCTCCTCCTGGTGCCTTAC	559	RT-PCR

		5′: TTGGTCCGCTTCTACTACCTG 3′: TGGATGGAGTCGATGGAAATCT	98	qPCR (PrimerBank ID: 26340698a1)

*Plcz1* (ζ1)	146149119	5′: CTCGCAGAAGCAAGATGGTTT 3′: GGCATGGGAAATCAAGTTTCTCA	109	RT-PCR (PrimerBank ID: 22550102a1)

#### RNA extraction, first-strand cDNA synthesis, and agarose gel electrophoresis

Animal euthanasia, tissue dissection and the two-step RT-PCR protocol were described in our previous publication (Sathyanesan et al., 2013), with minor modifications. Briefly, individual mice were euthanized with CO_2_ and olfactory turbinate tissue and positive control tissues including brain, spleen, testis were freshly dissected, homogenized, and total RNA was extracted. For first-strand cDNA synthesis, 500 ng of total RNA template was used and one aliquot (1 μl) of the cDNA reaction was used as the starting template for polymerase chain reaction (PCR). For PLC isozymes that were not expressed at these standard amounts, the cDNA synthesis and PCR steps were repeated with higher amounts of starting material at each step (1.5 μg total RNA and five aliquots of the cDNA reaction for each isozyme PCR). Additionally, PCR cycles for isozymes that were not expressed with the standard amount of 500 ng of total RNA were increased from 30 to 40 cycles. Templates from cDNA reactions without reverse transcriptase served as negative controls. Resulting amplicons were electrophoretically resolved on 2% agarose gels.

### REAL-TIME QUANTITATIVE POLYMERASE CHAIN REACTION

The qPCR and data analysis were carried out as previously described (Sathyanesan et al., 2013). Some of the primers used for RT-PCR were also used for qPCR since the amplicon size was in the range desirable for qPCR (**Table 1**).

### RNA *IN SITU* HYBRIDIZATION

RNA *in situ* hybridization protocol including solution making, tissue preparation, riboprobe synthesis, hybridization, and image acquisition have been described in detail in Sathyanesan et al. (2013). Briefly, RNase-free conditions were achieved using 0.1% diethyl pyrocarbonate (DEPC) treated solution and RNase Zap (Sigma). Digoxygenin (DIG) labeled riboprobes were synthesized from RT-PCR amplicons cloned into the pGEM-T-Easy vector (Promega, Fitchburg, WI, USA). We used the same PCR primers listed in **Table 1** for making PLCβ4, γ1, and γ2 RISH probes. Additionally, we adapted a pair of PLCβ4 primers from Allen brain atlas data portal: *in situ* hybridization data (5′: GCAGGTTATATCAGGGCAGTTC, 3′: CGCCTTCTCTAGGATTTTCTCA). Procedure for acquiring adult olfactory tubinate tissue from manually deboned noses can be further obtained from our recent video and publication (Dunston et al., 2013). For RISH analysis, we used 14 μm-thick MOE sections.

### STATISTICAL ANALYSIS

For comparison of Ca^2+^ responses, Student’s *t*-test statistical analyses were performed. *p* < 0.05 was considered to be statistically different. Bar graphs represent mean percent changes at the peak of the responses from resting Ca^2+^ levels ± SEM. The mean values were obtained by averaging all the responses in each set of experiments. For comparison of qPCR data of PLC isozyme expression levels, one-way ANOVA followed by Tukey’s *post hoc* analysis was performed. *p* < 0.05 was considered statistically significant. Bar graphs represent mean expression (*n* = 3 mice) of PLC isozyme transcripts normalized to expression of glyceraldehyde 3-phosphate dehydrogenase (*Gapdh)* transcript ± SEM.

## RESULTS

### THE PLC ACTIVATOR m-3M3FBS-INDUCED INTRACELLULAR Ca^2+^ INCREASES IN ISOLATED OSNs

**FIGURE 1:**
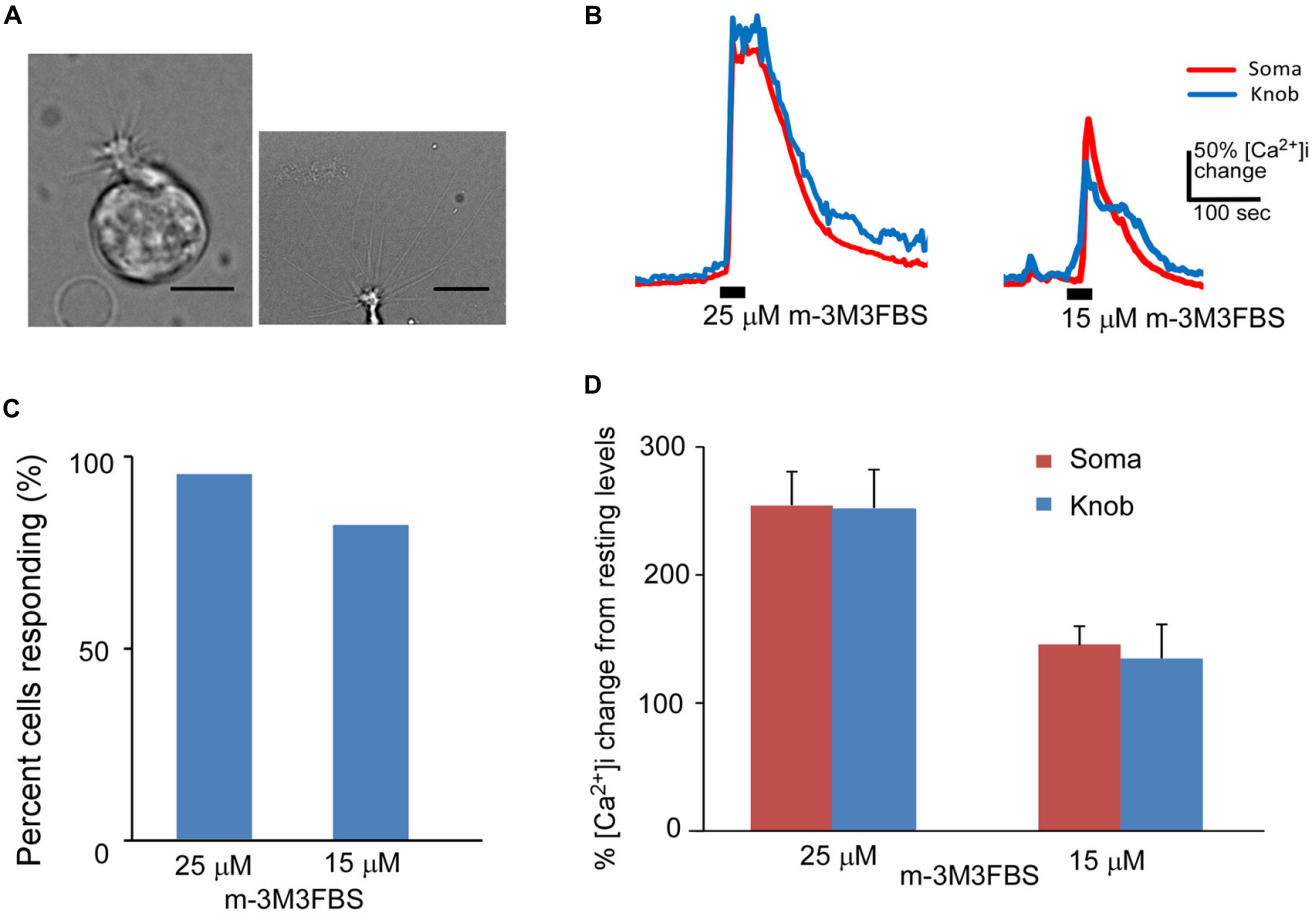
**Phospholipase C activator m-3M3FBS increases Ca^**2+**^ levels in isolated OSNs. (A)** Left: a representative image of an isolated OSN used for our recordings. Right: an image of the apical region of an isolated OSN, showing more than 10 long cilia emanating from a dendritic knob. Scale: 5 and 10 μm for left and right panels, respectively. **(B)** Representative Ca^2+^ response traces recorded from OSN soma (red) and dendritic knob (blue) to 25 and 15 μM m-3M3FBS in imaging experiments. Bars indicate the stimulation period of 35 s. **(C)** Percentage of OSNs responding to 25 μM (*n* = 47) and 15 μM (*n* = 32) m-3M3FBS. **(D)** Plot of averaged peak response amplitudes to 25 and 15 μM m-3M3FBS, respectively. The vertical scale bar in **(B)** and Y-axis in **(D)** stand for percent changes in the intracellular Ca^2+^ level ([Ca^2+^]_i_) measured from the resting Ca^2+^ level of the recorded region of the same cells (Mean ± SEM).

To investigate changes in intracellular Ca^2+^ levels mediated by direct PLC activation, we freshly dissociated OSNs from the MOE and stimulated them with the PLC activator m-3M3FBS in intracellular Ca^2+^ imaging. For our experiments, we selected OSNs that had multiple relatively long cilia. The lengths of the cilia varied, some of which were over 30 μm, indicating the OSNs we recorded were mature (**Figure 1A**, left panel: a typical isolated OSN; right panel: image with focus on cilia emanating from the knob). In addition, we also monitored OSN’s excitability by challenging randomly selected OSNs with either a high K^+^ (40 mM) bath solution and/or forskolin (3 μM). The high K^+^ solution induces an increase in intracellular Ca^2+^ levels by depolarizing the cell membrane, which consequently activates voltage-gated Ca^2+^ channels. Forskolin activates adenylyl cyclase, leading to activation of the canonical cAMP-mediated odor transduction pathway. All OSNs tested responded to high K^+^ and forskolin (Supplementary Figure 1), indicating that the OSNs we selected for our experiments were in healthy conditions with well-maintained morphology and excitability. For this study, we used a 40x oil objective lens, which enables us to reliably measure Ca^2+^ levels in the both dendritic knob and soma of individual OSNs. For the purpose of this study, we did not determine what odor receptors these isolated OSNs expressed. Because we randomly selected OSNs that were isolated from the entire olfactory turbinates, they most likely expressed different odor receptors.

We freshly made working solution of PLC activator m-3M3FBS from its stock solution (25 mM in DMSO) immediately before each experiment. Bath-application of m-3M3FBS resulted in a large increase in intracellular Ca^2+^ in both the knob and soma regions. **Figure 1B** shows typical traces of Ca^2+^ responses evoked by 25 and 15 μM m-3M3FBS, respectively for 35 s application. Approximately 93.6% (44 out of total 47 OSNs tested from 25 mice) and 81.3% (26 out of 32 OSNs tested from 13 mice) OSNs, respectively, responded to 25 and 15 μM m-3M3FBS (**Figure 1C**). There is no statistical difference between the percent responding cells to the two concentrations (*p* = 0.15, Fisher’s exact test, two-tail). **Figure 1D** presents the averaged changes in the Ca^2+^ levels induced by 35 s application of 25 and 15 μM m-3M3FBS, respectively, with significantly higher response amplitude observed at 25 μM (*p* < 0.05, two-tail *t*-test, *n* = 44 and 26 for 25 and 15 μM, respectively). These results indicate that PLC activity is present in a vast majority of mouse mature OSNs and that activation of PLC is a potent mechanism to increase intracellular Ca^2+^.

### SPECIFICITY OF THE PLC ACTIVATOR

**FIGURE 2:**
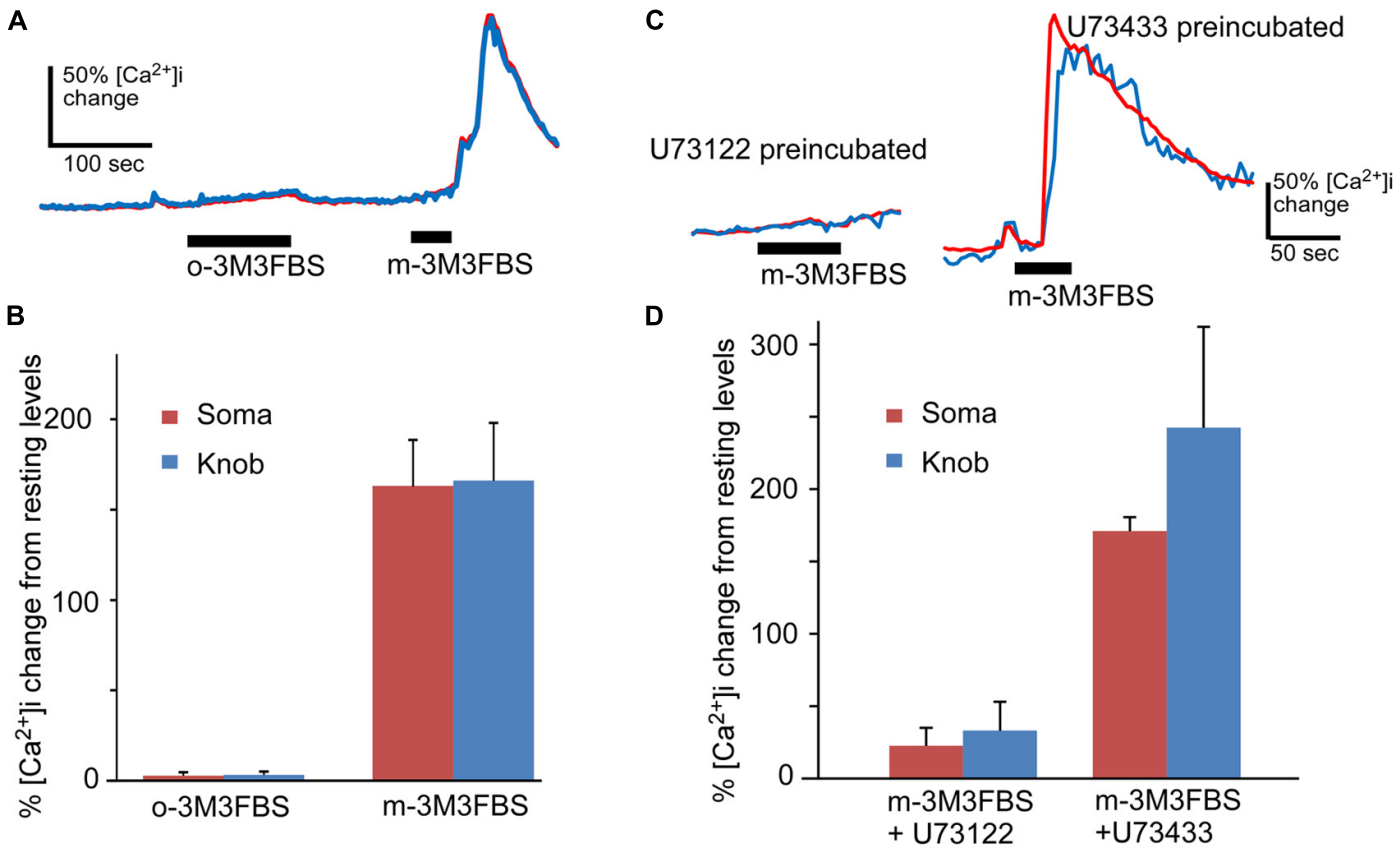
**m-3M3FBS specifically activates PLC in OSNs. (A)** Representative response records for o-3M3FBS, which is the inactive analog of m-3M3FBS, and m-3M3FBS at 25 μM in Ca^2+^ imaging experiment. o-3M3FBS failed to induce Ca^2+^ increase in the same cells tested responsive to m-3M3FBS. Bars indicate the stimulation period. **(B)** Average of peak responses to 25 μM o-3M3FBS and m-3M3FBS. The mean response amplitude value induced by o-3M3FBS is significantly smaller than the value induced by m-3M3FBS (*p* < 0.005, paired *t*-test, two-tail, *n* = 8). **(C)** Representative response records to 25 μM m-3M3FBS in OSNs after incubation with PLC inhibitor U73122 or its inactive analog U73433 (5 μM, each). **(D)** Averaged peak response values (Mean ± SEM) to 25 μM m-3M3FBS after pre-incubation of either U73122 (*n* = 8) or U73433 (*n* = 5), showing that U73122 significantly reduces the response to m-3M3FBS as compared to U73433 (*p* < 0.01, two-tail, *t*-test). Note the lengthened stimulation times for o-3M3FBS and for m-3M3FBS after U73433 to ensure their effects were recorded.

To test the specificity of m-3M3FBS in OSNs, we performed the same Ca^2+^ imaging experiment using the inactive analog o-3M3FBS. o-3M3FBS (25 μM) produced only a slow and very small Ca^2+^ increase in two out of eight OSNs tested even for longer application time (*n* = 4 mice), in contrast to the Ca^2+^ increase evoked by the active activator m-3M3FBS applied for 35 s after to the same cells (**Figure 2A**). The averaged response amplitude is plotted in **Figure 2B**, showing drastic differences in the response amplitude between these two analogs (*p* < 0.005, paired *t*-test, two-tail, *n* = 8). To further examine the specificity of the PLC activator in OSNs, we pre-incubated the OSNs with the PLC inhibitor U73122 (5 μM) for 5 min and monitored the effect of U73122 on m-3M3FBS (25 μM)-induced Ca^2+^ increases. The Ca^2+^ recording traces and the average response amplitude plot are shown in **Figures 2C,D**, respectively. U73122 suppressed the m-3M3FBS-induced responses amplitude even after longer m-3M3FBS application time (**Figure 2D**) and the percent responding cells (27.5% responding cells; three out of eight cells from 3 mice). For control, we repeated the experiment with the U73122 inactive analog U73433 (5 μM). All five cells tested with U73433 responded to m-3M3FBS. The difference in the response amplitude between U73122 and U73433 treatment is statistically significant (*p* < 0.01, *t*-test, two-tail, *n* = 8 and 5 for U73122 and U73433, respectively). Thus, under our experimental conditions, m-3M3FBS activates PLC in OSNs specifically.

Additionally, we examined the autofluorescence of m-3M3FBS because a previous study has indicated that the autofluorescence of m-3M3FBS may induce artifacts in the fluorescence measurements (Jansen et al., 2004). We measured ratiometrically the fluorescence intensity of the bath solutions before and after adding different concentrations of m-3M3FBS. Under our optical recording condition for Fura-2 ratio imaging, m-3M3FBS at 15 and 25 μM induced only 0.10 ± 0.04 and 0.17 ± 0.09% changes from the background fluorescence intensity (*n* = 11 and 18, respectively; Supplementary Figure 2). We also applied 100 μM m-3M3FBS and did not see any higher than 1% changes in the background intensity in the bath (data not shown). This result rules out the potential effect of m-3M3FBS autofluorescence on our results.

### INVOLVEMENT OF INTRACELLULAR Ca^2+^ STORES IN PLC ACTIVATION INDUCED Ca^2+^ INCREASES

**FIGURE 3:**
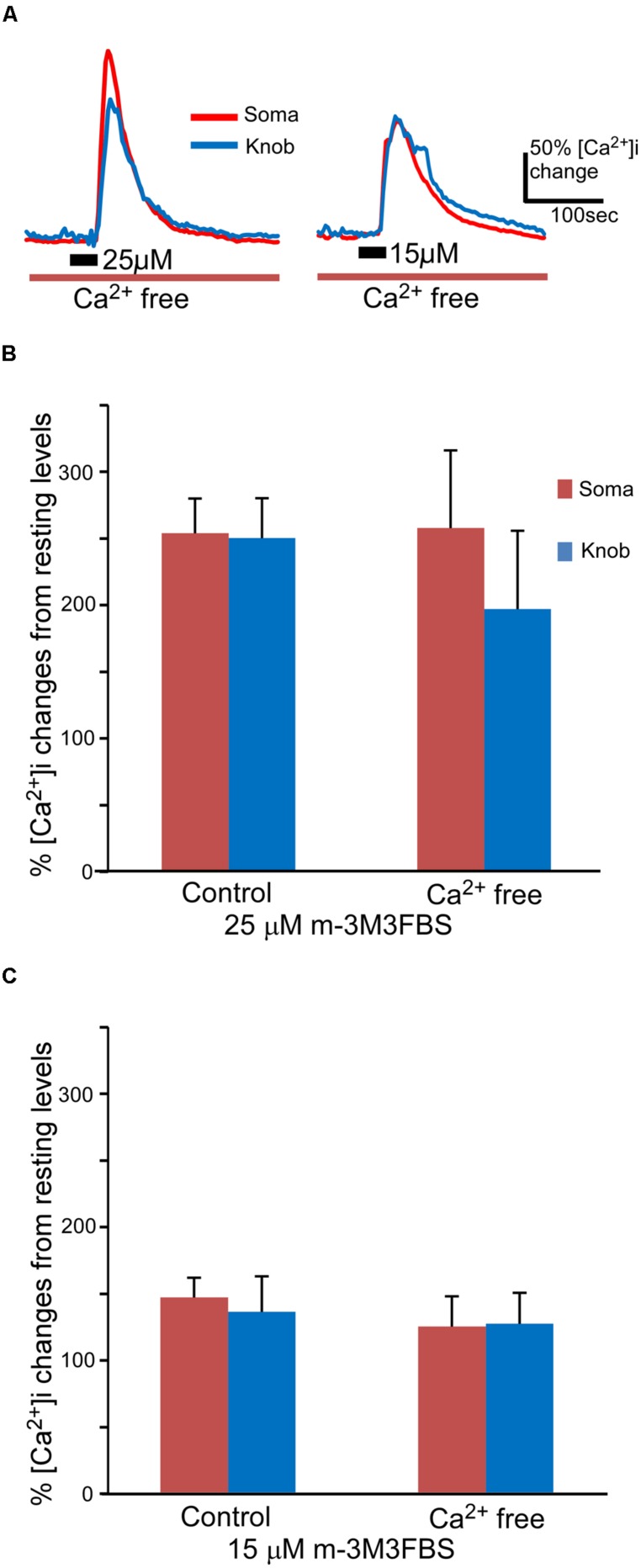
**m-3M3FBS induces intracellular Ca^**2+**^ increases primarily via internal Ca^**2+**^ release. (A)** Representative records of responses to 25 and 15 μM m-3M3FBS in Ca^2+^ free Tyrode’s saline. **(B,C)** Averaged peak response values to 25 μM **(B)** and 15 μM **(C)** m-3M3FBS in normal and Ca^2+^ free saline (*n* = 44 and 7 for 25 μM and *n* = 26 and 10 for 15 μM, respectively, Mean ± SEM).

Both second messenger IP_3_ and DAG produced as the consequence of PLC activation are capable of increasing intracellular Ca^2+^ via either internal or external sources in sensory receptor cells (Restrepo et al., 1990; Schild et al., 1995; Ogura et al., 1997; Lucas et al., 2003; Zhang et al., 2010). We removed extracellular Ca^2+^ from the bath solution to determine whether the external or internal Ca^2+^ source is responsible for the PLC-induced Ca^2+^ increases. To avoid shocking the cells, we first reduced extracellular Ca^2+^ levels by switching from the normal Tyrode’s saline to a nominal Ca^2+^ free saline and then replaced it with a BAPTA (150 μM)-containing Ca^2+^ free saline. m-3M3FBS was only applied once per cell for 35 s, since subsequent applications of m-3M3FBS often induced smaller responses as compared to the first response (data not shown). As shown in **Figure 3A**, m-3M3FBS at both 25 and 15 μM induced sizable intracellular Ca^2+^ increases in both soma and knobs in Ca^2+^-free bath solution. Also, similar to the m-3M3FBS-induced Ca^2+^ responses in normal Tyrode’s, high percent of OSNs tested responded to both 25 and 15 μM in Ca^2+^-free bath solution (7 of 7 cells and 10 of 12 cells, for 25 and 15 μM m-3M3FBS, respectively). There is no statistical difference between the percent responding cells to the two concentrations (*p* = 0.51, Fisher’s exact test, two-tail). In the responses produced by 25 μM m-3M3FBS, the averaged response amplitude measured in the soma in the Ca^2+^-free bath was very close to the value obtained in the normal Tyrode’s (**Figure 3B**). We did not find any statistical difference between the two values (*p* = 0.95, *t*-test, two-tail, *n* = 44 and 7 for normal and Ca^2+^ free Tyrode’s solutions, respectively). The average peak value measured from the knobs in the Ca^2+^ free solution was lower than that measured in the normal Tyrode’s solution (**Figure 3B**), but the difference was not statistically significant (*p* = 0.44, *t*-test, two-tail, *n* = 44 and 7 for control and Ca^2+^ free, respectively). When comparing the 25 μM m-3M3FBS-induced Ca^2+^ responses in Ca^2+^ free solution (**Figure 3B**, Ca^2+^ free), the difference between soma and knob region is statistically significant (*p* < 0.05, two-tail, paired *t*-test). In the responses produced by 15 μM m-3M3FBS, the average response amplitudes from both soma and knobs obtained in the Ca^2+^ free solution were only slightly reduced as compared to the amplitude from those obtained in normal Tyrode’s (**Figure 3C**), and the difference was not statistically significant (*p* = 0.43 and 0.80 for soma and knob, respectively, *t*-test, two-tail, *n* = 26 and 10 for control and Ca^2+^ free solutions, respectively). These results indicate that PLC activation-induced increases in Ca^2+^ levels are primarily due to Ca^2+^ release from internal stores.

### ODOR MIXTURE-INDUCED Ca^2+^ INCREASES IN THE ABSENCE OF EXTERNAL Ca^2+^ AND INVOLVEMENT OF PLC

**FIGURE 4:**
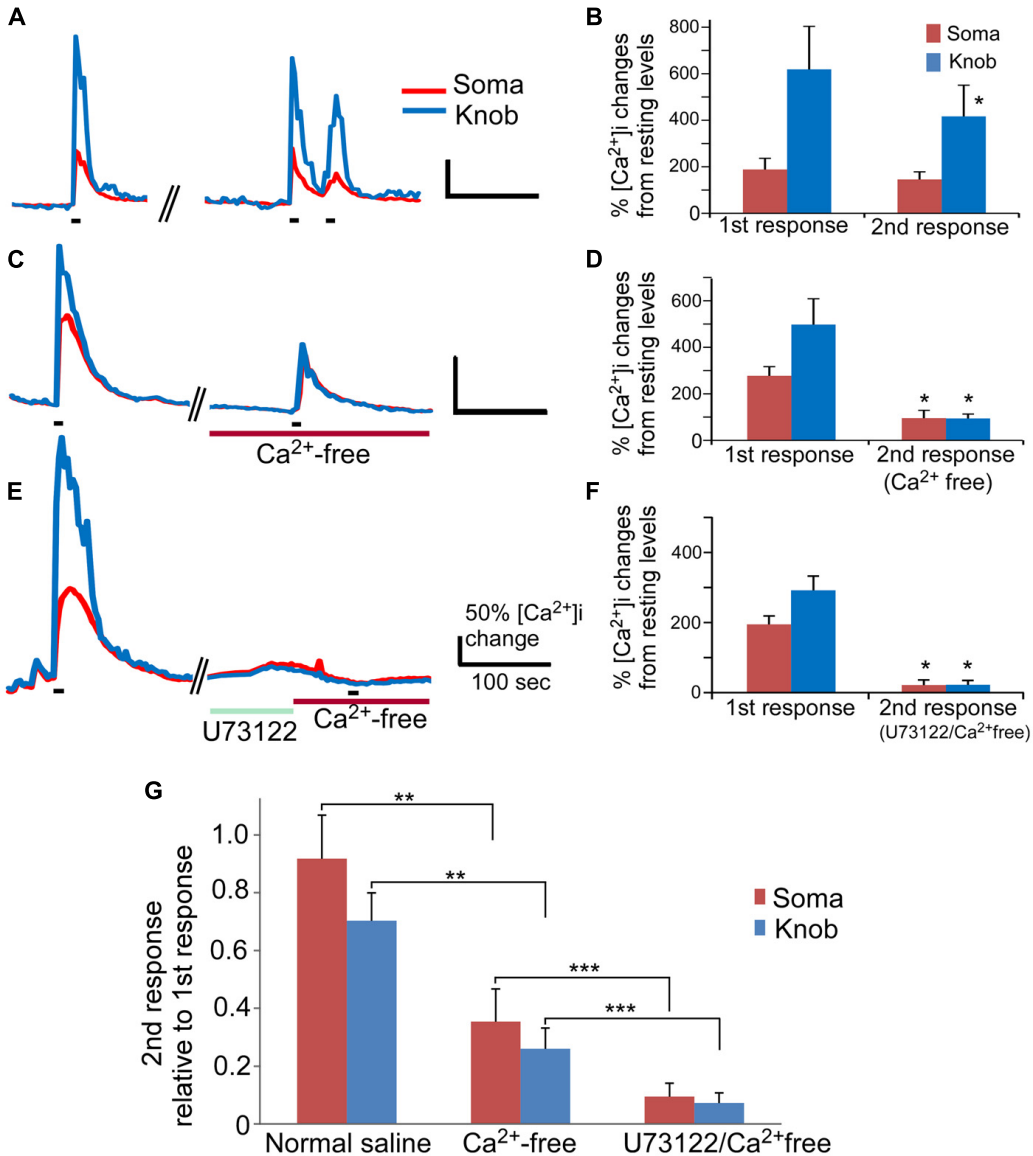
**The PLC-mediated Ca^**2+**^ increase contributes to an odor mixture-induced intracellular Ca^**2+**^ increases. (A,C,E)** Representative records of changes in Ca^2+^ levels from isolated OSNs in response to repetitive stimulation with an odor mixture. Red and blue color traces are obtained from the soma and dendritic knob regions, respectively. Black bars under the recording traces indicate 15 s stimulation. The first stimulation was applied in normal saline, followed by second stimulation in either normal saline **(A)**, or in Ca^2+^ free **(C)**, or in Ca^2+^ free after incubation with U73122 **(E)**. Scale bars in **(A,C,E)** 50% change in Ca^2+^ level from the resting level and 100 s. **(B,D,F)** Averaged peak Ca^2+^ responses from 1st to 2nd stimulation correlating to (**A,C,E;** *n* = 9, 6, and 7 cells, respectively). Asterisk denotes the averaged second responses are significantly different compared to the paired first responses from the same cells (two-tail, paired *t*-test, *p* < 0.05). **(G)** Comparison of the normalized second response values obtained either in normal, Ca^2+^ free or Ca^2+^ free plus U73122 saline. The relative second response values were calculated by normalizing the values of the second responses to the values of the first responses from the same cells. Note that statistically significant differences are found between the normal and Ca^2+^ free saline (two-tail, *t*-test, **p < 0.05) and between Ca^2+^ free and Ca^2+^ free saline plus U73122 pre-incubation (two-tail, *t*-test, ***p < 0.05).

Our results indicate that activation of PLC represents an important route to change Ca^2+^ levels in a high percentage of OSNs. However, the involvement of PLC in rodent odor transduction remains controversial. In mouse OSNs, Ca^2+^ increase-mediated by the canonical cAMP olfactory signaling pathway indispensably relies on Ca^2+^ influx through the CNG channels (Frings et al., 1995). We reasoned that, since the PLC activator-induced Ca^2+^ increases rely on internal Ca^2+^ stores, odor-induced responses would be detectable in Ca^2+^ free saline if the PLC plays a role in odor signal transduction. To examine the involvement of PLC in odor-induced responses, we stimulated OSNs with an odor mixture in the presence and absence of extracellular Ca^2+^. The odor mixture contains 12 individual odorants at 10 μM each (see Materials and Methods for the list of odorants) to increase the percent responding OSNs. Also, because odor responses attenuate over repeated stimulation and over time, we carefully paired the experiments to ensure data comparable. In control experiments, isolated OSNs were repetitively stimulated with the odor mixture (15 s stimulation duration, ∼240 s interval between 1st and 2nd stimulation), and the Ca^2+^ responses were recorded (**Figure 4A**). About 30% OSNs responded to odor mix stimulation in normal Tyrode’s containing 3 mM Ca^2+^ with a sharp increase in the intracellular Ca^2+^ levels (35 out 117 cells tested in 19 mice). In general, the peak response amplitude-induced by the odor mixture in the knob was higher than in the soma. When the odor mixture stimulation was repeated, the second response generally was smaller than the first response obtained from the same cells, which is consistent with previous reports (Maue and Dionne, 1987; Hegg and Lucero, 2004). In the soma, the averaged peak response value of the second response was 92% of the first response peak value (**Figures 4B,G,** normal saline). The differences between 1st and 2nd response was not significant (**Figure 4B,** *p* = 0.06, paired *t*-test, two-tail, *n* = 9). In the knob, the run-down was slightly faster with the average magnitude of second responses being about 75% of the value of first responses. The difference is statistically significant (**Figures 4B,G** normal saline; *p* < 0.05, paired *t*-test, two-tail, *n* = 9). When the odor mix was applied for the second time in the Ca^2+^-free saline containing 5 mM BAPTA (15 s stimulation duration, ∼270 s interval between 1st and 2nd stimulation), five out of six OSNs tested responded with a relatively small, but measurable Ca^2+^ increase in both the knob and the soma (**Figure 4C**). The amplitude values of the second response measured from the soma and the knob in the Ca^2+^-free solution were 35 and 26%, respectively, of the first response measured from the same region of the same cells in the normal saline (**Figure 4D**, *p* < 0.05 between 1st and 2nd responses for both soma and knobs, paired *t*-test, two-tail, *n* = 6). Interestingly, due to the more reduction in the knob response amplitude in the Ca^2+^ free saline, the average peak response values for both the soma and knob became similar and not statistically significant (traces in **Figure 4C** and plot in **Figure 4D**, *p* = 0.96, *t*-test, two-tail, *n* = 6). These results demonstrate that a small portion of the Ca^2+^ increase induced by the odorant mixture in the knob and soma is from internal Ca^2+^ stores.

We further examined the role of PLC in the odor mixture-induced Ca^2+^ release in Ca^2+^-free bath by using the PLC inhibitor U73122. Pre-incubation of U73122 (5 μM) significantly reduced or eliminated the Ca^2+^ response to the 15 s odor mixture stimulation in Ca^2+^-free saline with stimulation interval ∼540 s (traces in **Figure 4E** and average amplitude plot in **Figure 4F**) as compared to the response obtained without U73122 pre-incubation (**Figures 4C,D**). **Figure 4G** shows the second response amplitude relative to the value of the first response, showing significant difference of the second response amplitude between in Ca^2+^ free and in U73122/Ca^2+^ free for both soma and knob region (*p* < 0.05, *t*-test, two-tail, *n* = 6 and 7 for Ca^2+^ free and U73122/Ca^2+^ free, respectively). These data provide further evidence that Ca^2+^ release via PLC activation is partially responsible for the odorant mixture-induced Ca^2+^ increases measured from the OSN cell bodies and dendritic knobs.

### RT-PCR ANALYSIS OF GENE TRANSCRIPT EXPRESSION OF PLC ISOZYMES

**FIGURE 5:**
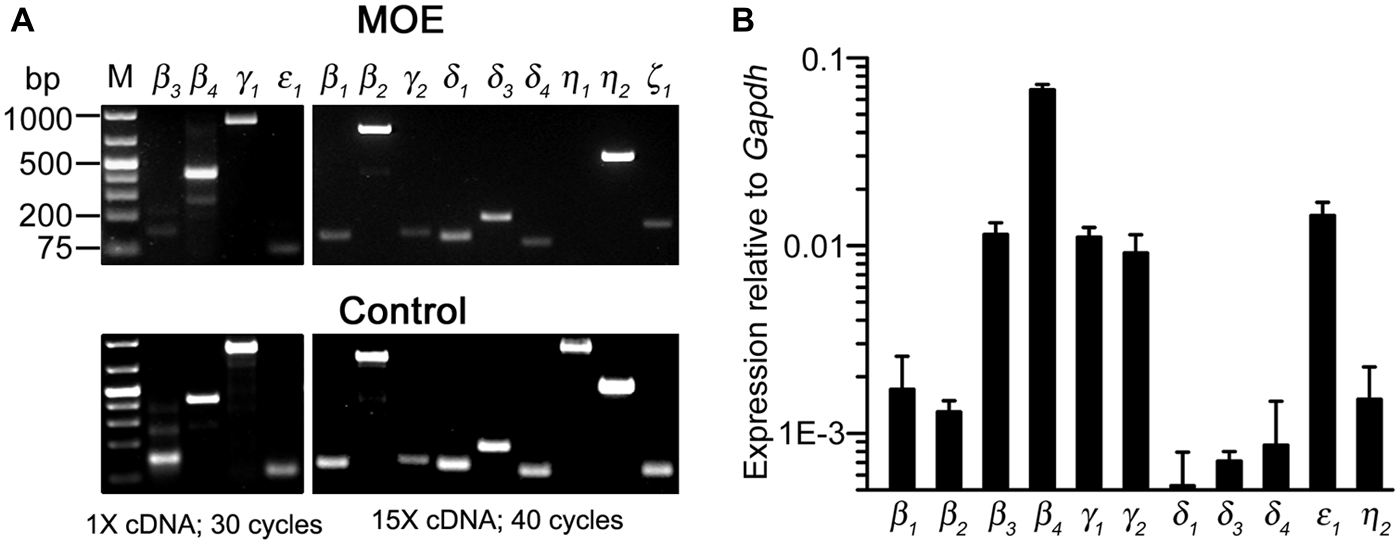
**Gene transcript expression of multiple PLC isozymes in olfactory turbinate tissue. (A)** RT-PCR analysis. Total RNA was extracted from olfactory turbinate tissue made up primarily of the MOE, and control tissues of brain for most of the isozymes except for β2 and ε1, for which spleen and testis RNA were used. Left: PCR products from a 30-cycle reaction that used 1 μl of the cDNA synthesis products (total 20 μl; started with 500 ng RNA). Right: PCR products from a 40-cycle reaction that used 5 μl of the cDNA synthesis products (total 20 μl; started with 1.5 μg RNA). **(B)** Real-time qPCR analysis. The expression levels are plotted relative to the expression of *Gapdh* reference gene, showing stronger expression of PLCβ3, β4, γ1, γ2, and ε1 as compared to PLCβ1, β2, δ3, δ4, η2 in the MOE.

To determine PLC gene transcript expression in the MOE, we used RT-PCR to probe all known mouse PLC isozymes. Using total RNA extracted from olfactory turbinate tissues in RT-PCR, we found a strong band of the PLCβ4 amplicant product and moderate or weak bands for PLC isozyme β3, γ1, and ε1 (**Figure 5A**, top left panel). Positive PCR products of these isozymes as well as other isozymes were obtained from control tissues including brain, spleen, and testis (**Figure 5A**, bottom panels). Because the lack of positive results for other PLC isozymes in the MOE preparations might be due to weak expression, we conducted RT-PCR experiments with increased amounts of RNA as starting template from 500 ng to 1.5 μg and an increased aliquot of the resulting cDNA from 1 to 5 μl for each PCR reaction. We also increased the number of PCR cycles from 30 to 40. Under such conditions, we observed additional positive bands for PLCβ1, β2, γ2 δ1, δ3, δ4, η2 (**Figure 5A**, top right panels). No product was found for PLCη1 and ζ1 although positive bands with correct sizes were found in control tissue (the size for ζ1 band in the MOE was incorrect). We repeated the experiments in RNA extract from three to six mice and confirmed the identity of all the amplicants by sequencing except δ1 due to the very low yield of the PCR product (Genewiz Inc.). These results indicate expression of multiple PLC isozymes in the olfactory turbinate tissue.

### REAL-TIME QUANTITATIVE PCR ANALYSIS OF PLC ISOZYME EXPRESSION

We next conducted qPCR to determine the relative expression levels of these isozymes in RNA extracted from the olfactory turbinate tissues. The expression level of a housekeeping gene glyceraldehyde phosphate dehydrogenase (*Gapdh*) was used for comparison. We found that PLC β3, β4, γ1, and ε1 were expressed at significantly higher levels than PLC β1, β2, δ1, δ3, δ4, and η2 (*F*_10,22_ = 104.4, *p* < 0.001 one-way ANOVA for 11 isozymes, α =0.05 for *post hoc* Tukey’s multicomparison; *n* = 3 mice; **Figure 5B**), with β4 expression being the highest. Additionally, qPCR data also indicate relatively high expression of γ2, which is comparable to the expression levels of β3, γ1 and ε1, although there is no significant difference from the isozymes expressed at lower levels (*F*_3,8_ = 1.18, *p* = 0.38, one-way ANOVA for the four isozymes, *n* = 3 mice). These quantitative results confirm the expression of multiple PLC isozymes in the olfactory turbinate tissues.

### RISH ANALYSIS ON CELL-TYPE SPECIFIC TRANSCRIPT EXPRESSION OF PLCβ4, γ1, AND γ2 IN THE MOE

**FIGURE 6:**
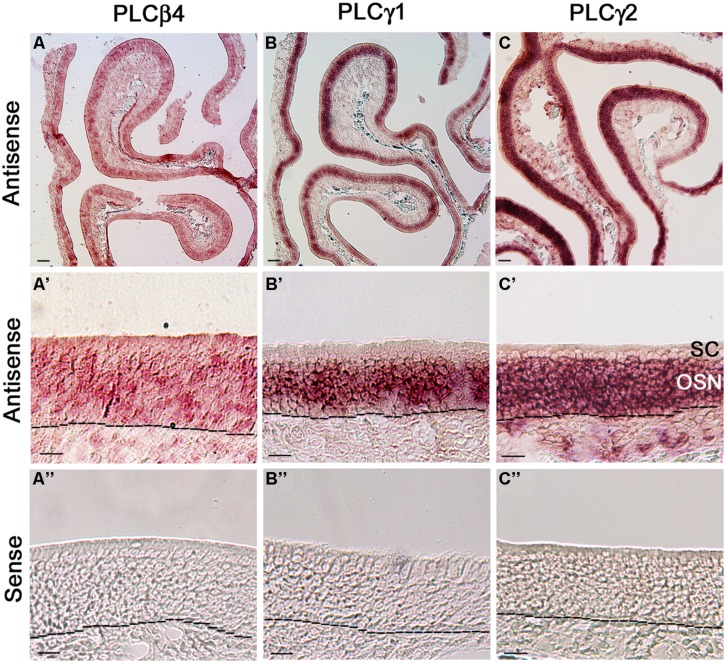
**RNA *in situ* hybridization analysis of PLCβ4, γ1, and γ2 isozyme expression in the MOE. (A,A’)** Lower and higher magnification images from MOE sections labeled with PLCβ4 antisense probes. RISH signal is present in various cell types of the MOE. as, antisense probe. Black dashed lines indicate basal lamina. **(A”)** Image from a section reacted with the PLCβ4 sense probe, showing absence of specific labeling. sen, sense probe. **(B,B’)** Lower and higher magnification images, showing PLCγ1 expression. The label is limited in the OSN layer. **(B”)** No labeling from the PLCγ1 sense probe. **(C,C’)** Lower and higher magnification images showing PLCγ2 expression in the MOE. OSN layer is strongly labeled. **(C”)** Image from the sense probe of PLCγ2. No labeling was found. SC, sustentacular/supporting cell layer; OSN, olfactory sensory neuron layer. Scale: **(A–C)** 50 μm; **(A’–C”)** 20 μm.

Because our qPCR data show that PLCβ4 is expressed at the highest level among all the isozymes and reportedly present in neurons of the cerebellum and retina (Lee et al., 1993; Roustan et al., 1995), we first probed its gene transcript expression in MOE coronal sections. We found that the RISH signal yielded from the PLCβ4 antisense probe is not confined to any particular layer or specific cell types in the MOE, although some cells in the OSN layer exhibited relatively stronger signal than their neighboring cells. Additionally, the antisense probe also positively labeled some cells in the lamina propria (**Figure 6A**, low magnification image, **Figure 6A**’ higher magnification image). The RISH experiment with the sense probe, which was conducted at the same time and under the same conditions, did not yield any apparent labels (**Figure 6A**”). To confirm this result, we repeated the RISH experiment using a different primer set, which sequence information was obtained from the Allen Brain Atlas data portal, to synthesize riboprobes. We obtained the same results (data not shown), indicating that the PLCβ4 mRNA transcripts are expressed in multiple cell types in the olfactory turbinate, which might result in a high expression level as indicated by our qPCR data.

We next examined the expression of two PLCγ isozymes because positive immunoreactivity for PLCγ1 is reportedly present in OSNs (Liu et al., 2006). We found that both PLCγ1 and γ2 antisense probes strongly label the OSN layers (**Figures 6B,C**: low magnification image, **Figure 6B’,C’**: higher magnification image, from the γ1 and γ2 antisense probes, respectively; **Figure 6B″,C″**: γ1 and γ2 sense probe labeling, respectively). Further close comparison between the two antisense labeling images, we found that the PLCγ1 signal is apparently restrict to the middle region of the MOE, where mature OSNs are located, while the γ2 signal is present in the entire OSN layer (dotted lines in the images represent the basal lamina), indicating that the isozyme is most likely expressed in both immature and mature neurons. Taken together, our RISH results confirm the expression of PLCγ1 and additionally, we show presence of PLCβ4 and γ2 in OSNs.

## DISCUSSION

We have investigated PLC-mediated activity and its isozyme expression in mouse OSNs using physiological and molecular approaches. Our results from single-cell Ca^2+^ imaging provide evidence that direct activation of PLC by m-3M3FBS leads to a large increase in intracellular Ca^2+^ in a vast majority of OSNs tested. This PLC-mediated Ca^2+^ increase is primarily from internal Ca^2+^ release since removal of extracellular Ca^2+^ has minimal effect on both the response amplitude and percent responding cells. We also showed that in the absence of extracellular Ca^2+^, the odor mixture-induced small Ca^2+^ response is mediated by PLC via internal Ca^2+^ release. Furthermore, we showed expression of multiple PLC isozymes and relatively higher levels of PLC β3, β4, γ1, γ2, and ε1 expression in the MOE as compared to other isozymes. Our RISH analysis confirms that OSNs express at least PLC β4, γ1, γ2 gene transcripts. Taken together, our results reveal the abundance of PLC-mediated activities and multiple isozyme transcript expression in mouse OSNs, providing basic knowledge for further investigation of PLC isozyme-mediated complex cellular signaling and regulations.

### PLC-MEDIATED ACTIVITIES VIA DAG AND DOWN-STREAM PROTEIN KINASE C

All 13 PLC isozymes identified to date are capable of catalyzing the hydrolysis of PIP_2_ to produce second messenger DAG and IP_3_ (Rhee, 2001). In mouse vomeronasal sensory neurons, DAG-mediated activation of TRPC2 is believed to play a key role in pheromone signal transduction (Spehr et al., 2002; Lucas et al., 2003; Zhang et al., 2010). Whether DAG directly activates ion channels in OSNs is not known currently. In our experiment, removal of external Ca^2+^ from the bath solution did not significantly reduce PLC-mediated intracellular Ca^2+^ increase. Thus, it is likely that the contribution of DAG-mediated Ca^2+^ influx via unknown ion channels to the overall PLC-mediated Ca^2+^ increase, if there is any, is minimal in mouse mature OSNs. Another major role of DAG is to activate protein kinase C (PKC), which is made up of a family of ten serine/threonine kinases (Takai et al., 1979; Kishimoto et al., 1980), and PKC related kinases (Mukai, 2003; Rykx et al., 2003). These kinases are known to be involved in cellular responses to environmental cues, cell proliferation, maturation, and regulation of gene expression in many cell types (Nishizuka, 1988). PKC has been studied in olfactory systems of various species. Bruch et al. (1997) found expression of multiple PKC gene transcripts in channel catfish olfactory tissue using RT-PCR (Bruch et al., 1997). In drosophila OSNs, PKC activation via the PLC pathway results in an enhancement of odor responses (Sargsyan et al., 2011) and altered behavioral preference to odorants (Tsunozaki et al., 2008). In mammals, odor stimulation induced phosphorylation of rat cilia proteins (Boekhoff et al., 1992). Pharmacological inhibition of PKC alters the odor-induced Ca^2+^ increases in isolated rat and human OSNs (Gomez et al., 2000). In addition to direct modulation of odor signaling, PKC also indirectly regulate signal output of OSNs by changing membrane excitability (Han and Lucero, 2006). Because DAG is the endogenous ligand for PKC and is produced primarily via PLC-catalyzed breakdown of inositol phospholipids, it is conceivable that PLC activity is common among OSNs of various species from these diverse effects of PKC. In our study, we found that over 90% of OSNs tested are activated by the PLC activator m-3M3FBS at 25 μM, a concentration commonly used in other investigations and at least three PLC isozymes expressed in the OSNs. These consistent results all point to the abundant presence of PLC in OSNs.

### PLC-MEDIATED INTRACELLULAR Ca^2+^ INCREASES VIA IP_**3**_

Unlike DAG, the role of IP_3_ in odor transduction has been investigated by various approaches (Schild and Restrepo, 1998). Certain odorants stimulate IP_3_ production in various species and in a heterologous expression system (Boekhoff et al., 1990; Breer et al., 1990; Restrepo et al., 1993; Ronnett et al., 1993; Klasen et al., 2010; Benbernou et al., 2011). In patch clamping and Ca^2+^ imaging experiments, IP_3_ activates a voltage-independent Ca^2+^ current and a non-selective cation current in the plasma membrane, leading to a large increase in the intracellular Ca^2+^ in the dendritic knob of *Xenopus laevis* OSNs (Schild et al., 1995). Similar results are also obtained from isolated OSNs of other species (Restrepo et al., 1992; Okada et al., 1994; Kashiwayanagi, 1996; Lischka et al., 1999; Kaur et al., 2001). The classic action of IP_3_, however, is to activate IP_3_ receptors located in the membrane of specialized endoplasmic reticula, i.e., Ca^2+^ stores and release Ca^2+^ internally (Rhee, 2001; Suh et al., 2008; Yang et al., 2013). In our study, removal of external Ca^2+^ did not significantly alter the Ca^2+^ responses to the PLC-activator m-3M3FBS when comparing soma to soma and knob to knob between normal and Ca^2+^-free Tyrode’s solutions. These results indicate that PLC-mediated Ca^2+^ increase in mouse OSNs primarily relies on internal Ca^2+^ release. Our results are different from the previous findings from frog and rat OSNs, where Ca^2+^ influx contributes primarily to the IP_3_-meditated Ca^2+^ increases (Restrepo et al., 1992; Okada et al., 1994; Lischka et al., 1999). Whether this discrepancy results from species difference is currently unknown. In addition, because we did not record from cilia, the primary site for odor transduction, we cannot rule out presence of IP_3_-mediated Ca^2+^ influx through ion channels in the plasma membrane responsible for signal transduction of select odorants.

In our experiment, 25 μM m-3M3FBS evoked significantly higher Ca^2+^ increase in the soma than in the knob in Ca^2+^-free Tyrode’s solution (**Figure 3B**). Such difference was not observed when stimulated with 15 μM m-3M3FBS (**Figure 3C**). Endoplasmic reticulum (ER), which presumably can serve as Ca^2+^ stores are present in the knob as indicated by the ER specific markers (Pietrobon et al., 2011). Due of the smaller size of the knob, ER in this region likely does not have the same capacity for Ca^2+^ storage, which might limit the amount of Ca^2+^ that can be released upon stimulation.

### POTENTIAL ROLE OF PLC IN REGULATING OSN ACTIVITY AND OUTPUT

According to the pioneering work of Maue and Dionne (1987), the large conductance Ca^2+^ activated K^+^ channels are the most frequently observed channels in both the knob and soma of mouse OSNs in patch clamp recordings and activation of these channels may also play a role in sensory adaptation in which the firing rate of OSNs is reduced during prolonged odor stimulation. PLC-mediated changes in intracellular Ca^2+^ levels potentially can regulate these channels and activation of PLC can be through purinergic receptors via Gq/G11 (Hegg et al., 2003; Verkhratsky, 2005; Yu and Zhang, 2014). In our study, we found expression of PLCβ4 using RISH and PCR. PLCβ4 can be activated via Gq/G11, which is reportedly present in OSNs (Menco et al., 1994).

### ODOR MIX-INDUCED Ca^2+^ INCREASE IN THE ABSENCE OF EXTERNAL Ca^2+^

In our Ca^2+^ imaging experiment, we observed a rapid increase in intracellular Ca^2+^ when stimulated with the odorant mixture in normal Tyrode’s. When the external Ca^2+^ is removed, the odorant response amplitude is significantly reduced, suggesting that the Ca^2+^ increase largely resulted from Ca^2+^ influx. Our results are consistent with the current view on the dominant role of cAMP pathway in rodent odor transduction. In this canonical pathway, odor stimulation leads to an increase in the intracellular cAMP level, leading to the opening of CNG channels and Ca^2+^ influx, which subsequently activates the Ca^2+^-activated Cl^-^ channels for further membrane depolarization via Cl^-^ eﬄux (Schild and Restrepo, 1998). However, in the absence of external Ca^2+^ (Ca^2+^ free saline plus 5 mM BAPTA) the odor mix still induced a small Ca^2+^ increase in most OSNs tested and the PLC inhibitor U73122 eliminated this response, suggesting a role of PLC and internal Ca^2+^ release in odor-evoked Ca^2+^ increase. These results are in consistent with a recent report by Pietrobon et al. (2011), in which they investigated odor-induced Ca^2+^ responses in rat cultured OSNs. They found that odor-evoked increase in intracellular Ca^2+^ persists in the Ca^2+^-free Rigner’s solution, which is blocked by PLC inhibitor U73122 and this internal Ca^2+^ release is sufficient to stimulate cGMP production in most OSNs tested. Pathways linking odor stimulation to PLC activation is not established. One possibility is via G-protein Gβγ subunits which are dissociated from the Gα_olf_ upon odor stimulation that activates the canonical pathway. We recently found that the Gβ_1_γ_13_ dimer is the dominant Gβγ subunits expressing in the mature OSNs (Sathyanesan et al., 2013). It will be interesting to determine whether Gβ_1_γ_13_ contribute to PLC isozyme activation in OSNs.

Also, whether this small Ca^2+^ response originates from the cilia or the knob is not known. An elegant experiment by Leinders-Zufall et al. (1997) revealed Ca^2+^ wave propagating from cilia to the knob and soma following odor stimulation in dissociated salamander OSNs. Due to the small diameter of mouse OSN cilia, we did not monitor ciliary Ca^2+^ changes. Because multiple PLC isozymes can be expressed in a single-cell in a spatially restricted fashion, the PLC-mediated events in cilia, if there is any, might not be the same as in the knob and soma.

### EXPRESSION OF MULTIPLE PLC ISOZYMES IN THE MOE

Our PCR experiments show that the olfactory turbinate tissue, which is made up mostly of MOE, expresses multiple PLC isozymes at various levels. This level of complexity is not surprising, given the diverse roles of PLC isozymes and presence of multiple cell types in the MOE. Previously, Liu et al. (2006) found expression of PLCβ1–3 and γ1 in the membrane fraction of Odora culture cells and rat MOE using Western blotting. They also confirmed the expression of PLCβ1–3 in rat OSNs and their axon bundles using immunolabeling. In our qPCR analysis, we found that the gene transcript expression levels of PLCβ1 and β2 are significantly lower than those of PLCβ3 and β4. In RISH analysis, we found expression of PLCβ4 transcript in the OSNs as well as other cell types of the MOE. Because PLCγ1 gene transcript was detected in sustentacular cells (Bruch et al., 1995), we also performed RISH analysis on the two PLCγ isozymes. In consistent with our qPCR results, our RISH data clearly show the expression of PLCγ1 and γ2 in the OSNs. Therefore, multiple PLC isozymes are expressed in mouse OSNs, supporting our findings in Ca^2+^ imaging.

The specific functions of these PLC isozymes in OSNs are yet to be determined. It is well known that mechanisms activating and regulating these isozymes are different. For example, PLCβ3 is activated through both Gα_q_ and Gβγ. PLCβ4 is activated by Gα_q_ and is insensitive to Gβγ. PLCγ1 and γ2 are activated via receptor tyrosine kinases, in contrast to the PLCβ group (Kamat and Carpenter, 1997; Carpenter and Ji, 1999). We recently found that the Gβ_1_γ_13_ dimer is the dominant Gβγ subunits expressing in the mature OSNs (Sathyanesan et al., 2013). It will be interesting to determine whether Gβ_1_γ_13_ contributes to PLC isozyme activation in OSNs.

### THE USE OF PLC ACTIVATOR AND ITS SPECIFICITY

In this study, we used the PLC activator m-3M3FBS to directly activate PLC because isozyme-specific activators and inhibitors are not available commercially. m-3M3FBS activates all PLC isozymes and has been used in studies of neurons, immune cells, sensory epithelial cells, and various cancer cells. Results of these studies have shown that m-3M3FBS induces intracellular Ca^2+^ release, IP_3_ production and reduction of PIP_2_, consistent with the expected outcome of PLC activation (Bae et al., 2003; Clapp et al., 2006; Li et al., 2009; Fujita et al., 2013). However, non-specific effects (Krjukova et al., 2004) and auto-fluorescence have also been reported (Jansen et al., 2004). Because there is no published result using m-3M3FBS in OSNs, we conducted stringent experiments to determine its specificity by: (1) pairing m-3M3FBS with its inactive analog o-3M3FBS; (2) using PLC inhibitor U73122 to block the m-3M3FBS responses, which was also paired with the inactive analog U73433; (3) determining Ca^2+^ source responsible for the m-3M3FBS-evoked Ca^2+^ increases; and (4) testing the auto-fluorescence of m-3M3FBS in the bath solution. Our results clearly demonstrate that m-3M3FBS specifically activates PLC in OSNs under our experimental conditions.

## CONCLUSION

In summary, we have provided strong physiological and molecular evidence for PLC-mediated changes in intracellular Ca^2+^ and expression of multiple PLC isozyme gene transcripts in a vast majority of mouse OSNs. Because the PLCβ4, γ1, and γ2 that we found in the OSNs can be activated via various cell surface receptors and intracellular Ca^2+^ influence a variety of cellular activities, we expect diverse and complex roles of PLC isozymes in OSNs. Our study therefore is significant in providing basic knowledge of PLC isozymes for future investigation, especially in the area of regulation of olfactory activities.

## AUTHOR CONTRIBUTIONS

Steven A. Szebenyi performed most of the Ca^2+^ imaging experiment. Tatsuya Ogura and Steven A. Szebenyi designed Ca^2+^ imaging experiments and performed data analysis. Tatsuya Ogura drafted some Ca^2+^ imaging results. Aaron Sathyanesan and Abdullah K. AlMatrouk performed RT-PCR, qPCR, and RISH experiments. Aaron Sathyanesan, Abdullah K. AlMatrouk, and Tatsuya Ogura performed qPCR data analyses. Justin Chang performed some Ca^2+^ imaging and data analysis. Weihong Lin conceived, supervised the project and drafted most of the manuscript. All authors read, edited and approved the manuscript.

## Conflict of Interest Statement

The authors declare that the research was conducted in the absence of any commercial or financial relationships that could be construed as a potential conflict of interest.
