# Supplementary material for: Increases in intracellular calcium via activation of potentially multiple phospholipase C isozymes in mouse olfactory neurons
Source: Front Cell Neurosci. 2014 Oct 21;8:336. doi: 10.3389/fncel.2014.00336 (PMC4204526; doi:10.3389/fncel.2014.00336)
Supplement: Supplementary file 1 [file Presentation_1.PDF]

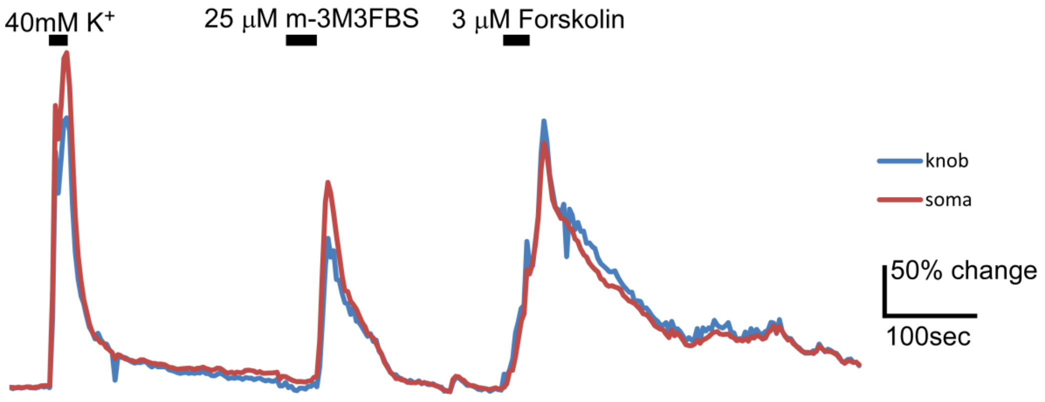

Supplementary figure1 A representative record from a mouse olfactory sensory neuron showing increases in intracellular Ca^{2+} levels in the soma (red trace) and dendritic knob (blue trace) in response to bath application of high K^{+} (40 mM) saline, the PLC activator m-3M3FBS (25 µM), and adenylyl cyclase activator forskolin (3 µM). Black bars on the top of each response indicate stimulus time.

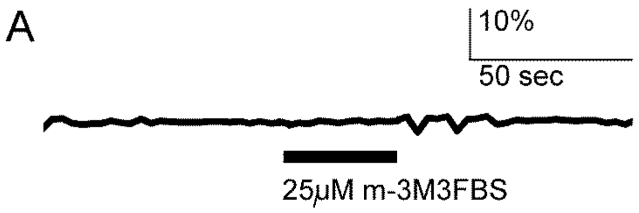

B

m-3M3FBS	Background change (%)
15 µM	0.10±0.04, n=11
25 µM	0.17±0.09, n=18

Supplementary figure2 A: A representative record from background region of a fluorescence image showing <0.5% changes in the fluorescence intensity during m-3M3FBS (25 µM) application (black bar). **B:** Mean peak values of fluorescence level changes in solution (cell-free background regions) during application of m-3M3FBS (15 and 25 µM).
